# Using Network Pharmacology to Explore the Mechanism of Peach Kernel-Safflower in the Treatment of Diabetic Nephropathy

**DOI:** 10.1155/2021/6642584

**Published:** 2021-02-01

**Authors:** Jingxue Han, Xinwei Wang, Jingyi Hou, Yu Liu, Peng Liu, Tingting Zhao

**Affiliations:** ^1^Beijing Key Lab for Immune-Mediated Inflammatory Diseases, Institute of Clinical Medical Sciences, China-Japan Friendship Hospital, Beijing 100029, China; ^2^Heilongjiang Academy of Chinese Medical Sciences, Harbin 150036, China; ^3^School of Chinese Materia Medica, Beijing University of Chinese Medicine, Beijing 100029, China; ^4^Shunyi Hospital, Beijing Traditional Chinese Medicine Hospital, Beijing 101300, China

## Abstract

**Objective:**

The mechanism of peach kernel-safflower in treating diabetic nephropathy (DN) was investigated using network pharmacology.

**Methods:**

Network pharmacology methodology was applied to screen the effective compounds of peach kernel-safflower in the SymMap and TCMSP databases. Potential targets were then screened in the ETCM, SEA, and SymMap databases to construct a compound-target network. This was followed by screening of DN targets in OMIM, Gene, and GeneCards databases. The common targets of drugs and diseases were selected for analysis in the STRING database, and the results were imported into Cytoscape 3.8.0 to construct a protein-protein interaction network. Next, GO and KEGG enrichment analyses were performed. Finally, Schrödinger molecular docking verified the reliability of the results.

**Results:**

A total of 23 effective compounds and 794 potential targets resulted from our screening process. Quercetin and luteolin were identified as the main effective ingredients in peach kernel-safflower. Furthermore, five key targets (VEGFA, IL6, TNF, AKT1, and TP53), AGE-RAGE, fluid shear stress and atherosclerosis, IL-17, and HIF-1 signaling pathways may be involved in the treatment of DN using peach kernel-safflower.

**Conclusions:**

This study embodies the complex network relationship of multicomponents, multitargets, and multipathways of peach kernel-safflower to treat DN and provides a basis for further research on its mechanism.

## 1. Introduction

Diabetic nephropathy (DN) is the most common and serious microvascular complication of diabetes [1]. With the increasing prevalence of diabetes, DN is now the major cause of end-stage renal disease [2–4]. At present, the therapeutic effect of western biomedicine for DN is not ideal. Conventional antihyperglycemic and antihypertensive treatments are not optimal for improving DN.

Traditional Chinese medicine (TCM) has long been used to treat diabetes and its complications [5]. TCM theory holds that DN is one of the results of blood stasis in that blood does not flow or circulate smoothly through the vessels. Almost all patients with DN have the pathologic state of blood stasis and show symptoms such as subcutaneous ecchymosis and petechiae. Therefore, one of the strategies for TCM to treat DN is to promote blood circulation and remove stasis.

Peach kernel (*Prunus persica* (L.) Batsch) and safflower (*Carthamus tinctorius* L.), two Chinese herbal medicines, which were first mentioned in the Qing dynasty medical textbook, *Golden Mirror of Medicine* (*Yi Zong Jin Jian*), and traditionally are used to promote blood circulation and remove blood stasis. The active chemical components of peach kernel are lipids, glycosides, sugars, and proteins. Its TCM functions are promoting blood circulation, removing blood stasis, and moistening intestine. Safflower mainly contains flavonoids, fatty acids, pigments, and phenolic acid. Its functions are promoting blood circulation to relieve pain and swelling [6, 7]. When paired, the effects of the two medicines are synergistic. However, the molecular mechanisms of their combined actions still lack systematic understanding.

**Figure 1:**
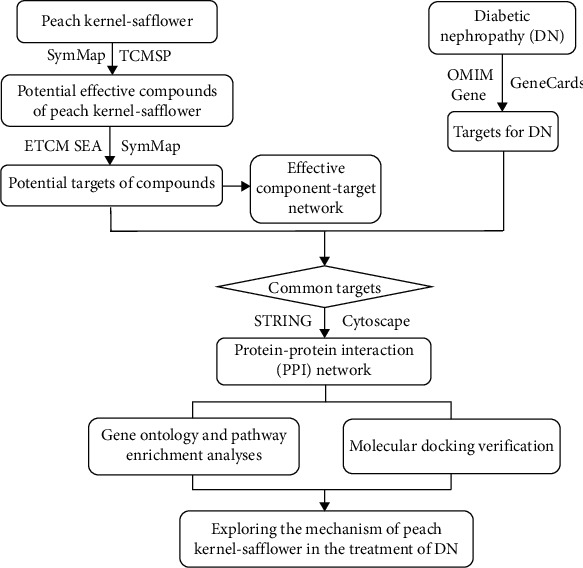
Flow diagram of the pharmacology-based study of peach kernel-safflower used in treating DN.

Network pharmacology explains the occurrence and development of diseases from the perspective of system biology and biologic network balance. It has the advantages of integration and systemization and is an effective method for studying the complex relationship between Chinese medicine and diseases [8, 9]. In this study, network pharmacology methods were used to clarify the potential mechanisms of peach kernel-safflower treatment of DN and provide a basis for subsequent pharmacologic experimental research (Figure 1).

## 2. Materials and Methods

### 2.1. Screening of Compound Components

The key words “peach kernel” and “safflower” were used to retrieve the compound components in the SymMap database (http://www.symmap.org) and in the Traditional Chinese Medicine Systems Pharmacology Database and Analysis Platform (TCMSP) database (https://tcmspw.com/tcmsp.php). The screening criteria were oral bioavailability (OB) ≥ 30% and drug − like (DL) ≥ 0.18.

### 2.2. Construction of the Component-Target Network

The targets of the compounds were searched through the Encyclopedia of Traditional Chinese Medicine (ETCM) database (http://www.nrc.ac.cn:9090/ETCM/index.php/Home/Index/index.html), the SymMap database, and the Similarity Ensemble Approach (SEA) database (http://sea.bkslab.org). The uniprot ID of the target was then searched through the Universal Protein Resource (UniProt) database (https://www.uniprot.org), with the species defined as “Homo sapiens.” All gene names were assigned their official gene symbol. Targets that did not meet the screening criteria were removed. Next, the network mapping software Cytoscape 3.8.0 (http://www.cytoscape.org) was used to construct networks for the compounds and their targets. In the network, a node represents a target, gene, molecule, or protein, and the connections between nodes represent the interactions between the targets, genes, molecules, or proteins. The “degree” value of a node represents the number of connections between the nodes in the network; the larger the value, the more likely the target is to become the key target of compounds.

### 2.3. Acquisition of Disease Targets

The key word “diabetic nephropathy” was searched in the Online Mendelian Inheritance in Man (OMIM) database (https://omim.org), GeneCards database (https://www.genecards.org), and Gene database (https://www.ncbi.nlm.nih.gov/gene) to obtain the disease targets.

### 2.4. Construction and Analysis of the Protein-Protein Interaction (PPI) Network

Potential targets of the retrieved compounds and disease targets were intersected, and the overlapping targets were selected and imported into the STRING database (https://string-db.org) to obtain the protein interaction relationship. The results were then imported into Cytoscape to construct and analyze the interaction network.

### 2.5. Screening of Core Clusters and Key Targets

Cytoscape plugin MCODE was applied for cluster analysis, and the filter conditions were set as Degree Cutoff: 2, *k*-core: 2 to select the core cluster with the closest relationship in the network. Then, the plugin CytoHubba was applied to analyze the PPI network and core cluster to obtain the network topology parameters. Targets shared by both the PPI network and core cluster with high degree were selected as the key targets, which were retrieved in the DisGeNET database (http://www.disgenet.org/search) to obtain the protein classes of the key targets.

### 2.6. Gene Ontology and Pathway Enrichment Analyses

The Gene Ontology (GO) database (http://geneontology.org) includes various functions of genes including biological process (BP), molecular function (MF), and cellular component (CC) and can be applied to the analysis of potential biologic molecular mechanisms. The KEGG database (https://www.kegg.jp) is used to identify biologic functions and candidate targets. In this study, ClusterProfiler (https://bioconductor.org/packages/release/clusterProfiler.html) in R package was applied to GO functional annotation and KEGG pathway analysis, and the enrichment analysis results were visualized.

### 2.7. Molecular Docking Verification

The Ligand Docking module in the Schrödinger chemical simulation platform (Schrödinger, New York, NY, USA) was used to verify the reliability of the results, and the bonding activity of the compound to the key targets was evaluated by the docking score. The structures of all the compounds were downloaded from the PubChem database (https://pubchem.ncbi.nlm.nih.gov/), and the three-dimensional structures of the key targets were downloaded from the Protein Database (PDB) database (http://www.rcsb.org/pdb/home/home.do). The higher the absolute value of the docking score, the stronger the binding ability of small molecules to protease targets.

## 3. Results and Analysis

### 3.1. Compound Screening

A total of 81 compounds were screened in the SymMap and TCMSP databases. Among the compounds, 15 in peach kernel and 10 in safflower met the screening criteria. There are two common compounds, so a total of 23 compounds were eventually included in the follow-up study (Table 1).

### 3.2. Target Prediction and Network Analysis of Compounds

Potential targets of compounds through ETCM, SymMap, and SEA databases were searched, and 794 targets of peach kernel-safflower were obtained after deleting duplicates.

**Figure 2:**
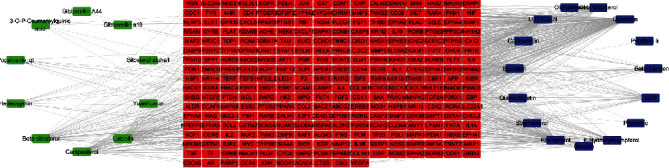
Effective component-target network. Green nodes represent compounds of peach kernel, blue nodes represent compounds of safflower, and red nodes represent predicted targets.

Using Cytoscape, we constructed a network relationship among compounds and predicted targets (Figure 2). The resulting network included 307 nodes and 794 interaction edges. The degree values of compounds in the compound-target network were then obtained (Table 2). Quercetin has 146 potential targets, followed by luteolin with 96, and kaempferol with 92. These higher-degree compounds are likely to be involved in treatment of DN by peach kernel-safflower.

### 3.3. Results of Disease Target Retrieval

With “diabetic nephropathy” as the keyword, a combined total of 524 disease targets were found in the OMIM, Gene, and GeneCards databases after deleting duplicates.

### 3.4. Screening of Drug-Disease Targets

The intersections of potential targets of peach kernel-safflower and disease targets resulted in 65 potential treatment targets for DN.

### 3.5. PPI Network of Peach Kernel-Safflower in the Treatment of DN and Key Target Analysis

**Figure 3:**
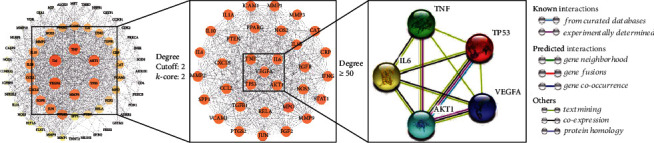
Network diagram of the PPI network, core clusters, and key targets: (a) PPI network; (b) core clusters; (c) key targets.

The PPI network was mapped using common potential targets of peach kernel-safflower and DN, consisting of 64 nodes and 880 interaction edges (Figure 3(a)). CytoHubba plug-in was used to analyze the PPI network to obtain core clusters (Figure 3(b)) and key targets (degree ≥ 50). Five targets with the largest degree value, vascular endothelial growth factor A (VEGFA), interleukin-6 (IL-6), tumor necrosis factor (TNF), AKT serine/threonine kinase 1 (AKT1), and tumor protein p53 (TP53), involves signaling molecules, calcium-binding proteins, kinases, transfer/carrier proteins, transferases, and transcription factors. The network of key targets was constructed based on the STRING database (Figure 3(c)). In the network, the key targets interacted with each other through known (from curated databases and experimentally determined), predicted (gene neighborhood, gene fusions, and gene cooccurrence), and other (text-mining, coexpression, and protein homology) interactions.

### 3.6. GO and KEGG Enrichment Analysis

**Figure 4:**
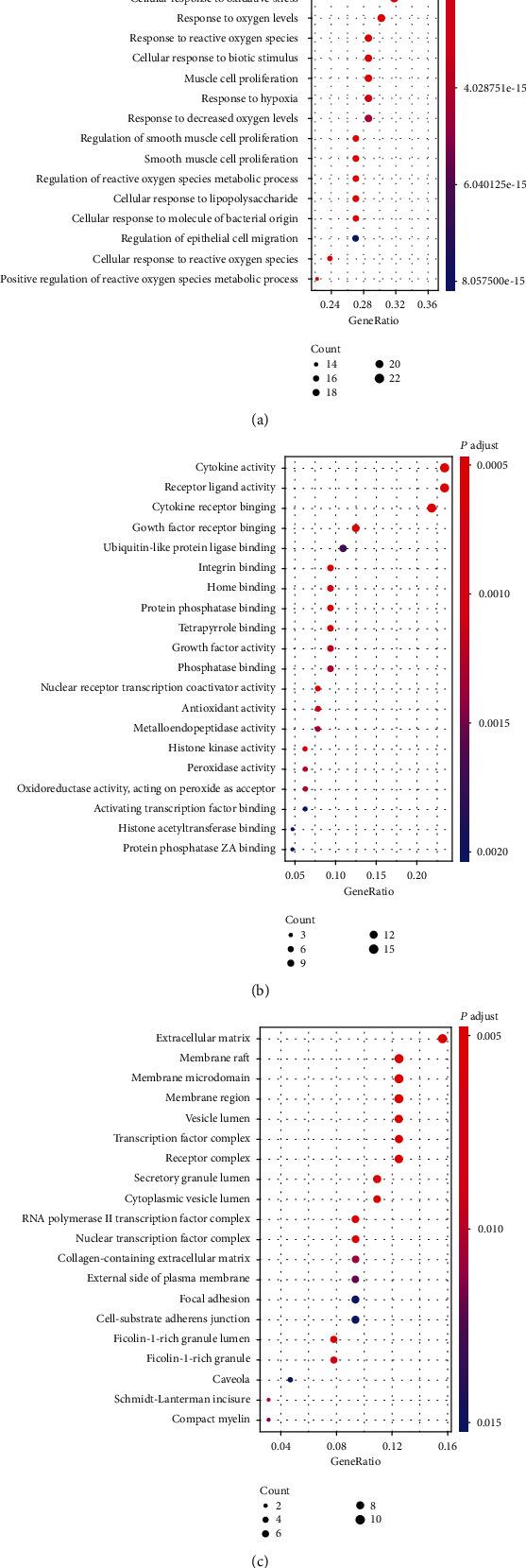
GO function enrichment analysis of potential targets from active ingredients in peach kernel-safflower: (a) biologic process; (b) molecular function; (c) cellular component.

GO functional annotation and KEGG pathway analysis were performed on 65 targets in the PPI network. The top 20 were then visualized as bubble charts (Figure 4). In the biologic process, peach kernel-safflower has great effect on lipopolysaccharides, molecules of bacterial origin, and oxidative stress (Figure 4(a)). At the molecular function level, drug components of peach kernel-safflower are mainly related to cytokine activity, receptor ligand activity, and cytokine receptor binding (Figure 4(b)). Targets in the cellular components are closely related to extracellular matrix, membrane raft, and membrane microdomain (Figure 4(c)).

**Figure 5:**
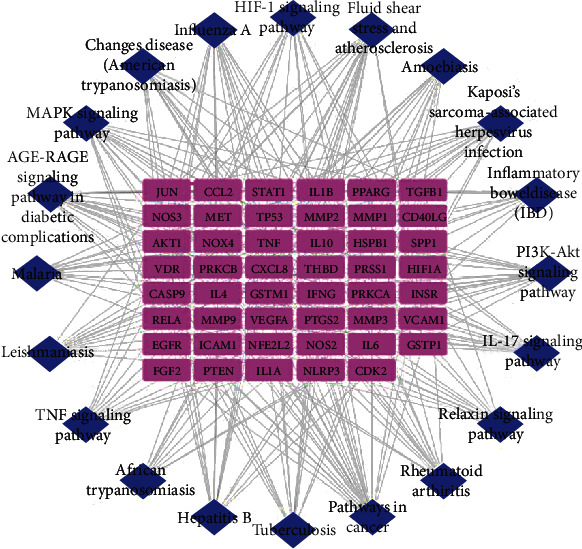
Target-KEGG pathway network. Blue nodes represent 20 KEGG pathways, and purple nodes represent common targets.

A total of 220 enrichment results were obtained by KEGG pathway analysis. The first 20 pathways were screened according to *P* adjust <0.05 (Figure 5) and consisted of 67 nodes and 289 interaction edges, which mainly involved signaling pathways such as advanced glycation end products-receptor for advanced glycation end product (AGE-RAGE) signaling pathway in diabetic complications, fluid shear stress and atherosclerosis, interleukin-17 (IL-17), hypoxia-inducible factor 1 (HIF-1), and TNF signaling pathway, thus indicating that the effective components of peach kernel-safflower might treat DN by acting on these pathways.

### 3.7. Verification of Results by Molecular Docking

**Figure 6:**
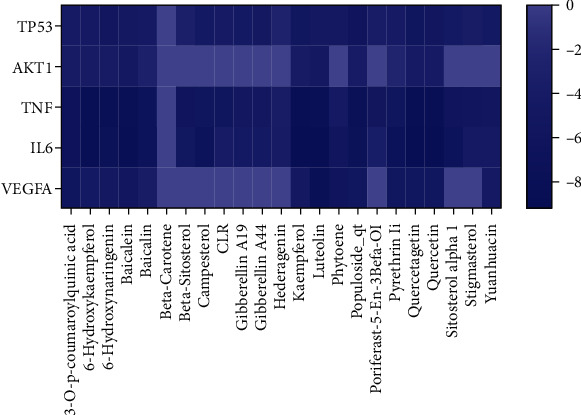
Heat maps of the docking scores of key targets combining with bioactive compounds in peach kernel-safflower.

The key targets VEGFA, IL6, TNF, AKT1, and TP53 were used for molecular docking with the effective compounds in peach kernel-safflower, and a heat map was drawn based on the results (Figure 6). All bioactive components of peach kernel-safflower had good binding with key targets, suggesting that peach kernel-safflower has a strong tendency as a therapeutic strategy for DN via these key targets.

**Figure 7:**
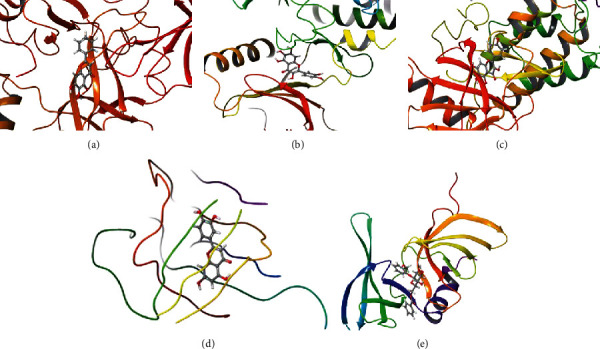
Molecular docking simulation of bioactive compound—key target: (a). AKT1—baicalein; (b) IL6—quercetagetin; (c) TNF—6-hydroxykaempferol; (d) VEGFA—luteolin; (e) TP53—populoside_qt.

Results showed that quercetagetin has a strong binding ability with IL6 (docking score = −9.216). Luteolin has a strong binding ability with VEGFA (docking score = −7.825). 6-Hydroxykaempferol binds with TNF (docking score = −8.362), baicalein binds with AKT1 (docking score = −4.858), and populoside_qt binds with TP53 (docking score = −6.002) (Figure 7).

## 4. Discussion

The Chinese medicine peach kernel-safflower is widely used in China to treat diabetic nephropathy (DN), but its mechanism of action is still not understood. In this study, based on the results of network pharmacology, we speculate that the compounds in peach kernel-safflower, quercetin and luteolin, are involved in the treatment of DN because of their high degree values (number of connections between the nodes in the network). Five key targets, VEGFA, IL6, TNF, AKT1, and TP53, which have high value in PPI network are also essential.

Quercetin is a bioflavonoid compound that can treat kidney disease through its antitumor, antioxidant, and antiproliferative effects [10]. The proliferation of glomerular mesangial cells is a common and prominent pathological change in the early stage of DN. Quercetin can inhibit the proliferation of mesangial cells in the early stage of DN through the Hippo signaling pathway, thereby delaying the process of DN [11]. Oral low-dose quercetin has appears to have a protective effect against DN in mice with hypercholesterolemia [12]. Luteolin possesses anti-inflammatory and antioxidant activities, which may protect the kidney against DN. Zhang et al. found that luteolin may inhibit the inflammatory response and oxidative stress by suppressing the activation of STAT3, so as to attenuate glomerulosclerosis and interstitial fibrosis in the DN mouse model [13].

VEGFA is a member of the vascular endothelial growth factor family [14]. One of the clinical features of DN patients is endothelial dysfunction [15]. Studies have found that mRNA levels of VEGFA in the glomeruli of patients with DN are significantly reduced. Loss of tubular epithelial cells leads to a decrease in VEGFA, which increases tubulointerstitial hypoxia by reducing the density of peritubule capillaries [16].

Inflammation is a prominent feature of DN [17]. IL-6 determines the stability of the chronic inflammatory environment throughout the DN process. The differentiation of renal monocytes into macrophages is an important factor in the establishment of the inflammatory environment and the production of fibrosis mediators, which is inseparable from IL-6 [18]. Studies have shown the involvement of IL-6 signaling in the process of DN [19–21]. Wu et al. demonstrated that tocilizumab, a humanized anti-IL-6 receptor antibody, has a protective effect against diabetic renal injury. Tocilizumab can reduce proteinuria and mesangial matrix accumulation and inhibit the inflammatory response, oxidative stress, and IL-6 signaling pathway in db/db mice [22].

TNF-*α* is an important regulator of the body's inflammatory response and immune function [23]. In inflamed kidney tissue, TNF-*α* can cause the release of a variety of inflammatory cytokines and amplify the cascade effect of inflammation [24, 25]. TNF-*α* can change the permeability of the glomerular basement membrane in patients with DN, participate in cell apoptosis and insulin signal transmission, and act synergistically on platelet activating factor to alter blood vessel structure. The rising level of TNF-*α* in the human body can cause abnormal glomerular structure and function [26].

Akt, also known as protein kinase B, is an important serine/threonine protein kinase in the intracellular signaling system and is the central link of the PI3K/Akt signaling pathway [27]. Abnormal expression and activation of Akt are involved in the pathologic process of diabetic renal fibrosis. Akt1 protein and mRNA are only slightly expressed in the kidney tissue of normal rats while in the kidney tissue of DN rats, the expression of Akt1 protein and mRNA is significantly increased [28, 29]. Pharmacologic and genetic approaches targeting p53 attenuated expression of related genes and reduced the fibrosis response, confirming the involvement of p53 in renal disease [30].

Through this network pharmacology study, we found that peach kernel-safflower in treating DN mainly involves the AGE-RAGE signaling pathway in diabetic complications, fluid shear stress and atherosclerosis, IL-17, HIF-1, and TNF signaling pathways. The AGE-RAGE signaling pathway in diabetic complications has been widely studied. Investigations have shown that AGE is the main environmental factor for diabetic vascular cell disorders, and RAGE is the main genetic factor that responds to it [31]. The binding of receptors for AGE (RAGE) and its ligands can cause oxidative stress and chronic inflammation of the kidney tissue, thereby causing the aberration of the kidney structure and ultimately the loss of kidney function [32, 33]. Sharma et al. have shown that AGE-RAGE interaction increases the expression of inflammatory cytokines, TGF-*β*, NF-*κ*B, and fibronectin, which promotes the progression of diabetic nephropathy [34].

HIF-1 is an oxygen-regulated transcriptional activator that is involved in physiology and disease pathogenesis [35]. In diabetes, hypoxia affects the kidneys. And HIF-1 is involved in hypoxia-induced tubulointerstitial fibrosis. Therefore, inhibiting the expression of HIF-1*α* protein may contribute to renal protection in DN [36]. Wang et al. found that HIF-1*α* is upregulated in DN patients, which may provide a clinical basis for the contribution of HIF-1*α* in the development of DN [37].

IL-17 is an inflammatory cytokine mainly produced by activated T cells [38]. IL-17 promotes activation of T cells and stimulates epithelial cells, endothelial cells, and fibroblasts to produce a variety of cytokines, thereby leading to inflammation [38]. Activation of the interleukin IL-17 pathway produces inflammatory cytokines, which are present in a variety of kidney diseases [39]. The inflammatory response and remodeling related to tissue damage and glomerulosclerosis in DN are closely related to IL-17 [40]. It is reported that low-dose recombinant IL-17A is a promising treatment for DN [41].

## 5. Conclusion

Network pharmacology explains the occurrence and development of diseases from the perspective of system biology and biological network balance [42]. We used its holistic and systematic advantages to explain the mechanism of action of the traditional Chinese medicine peach kernel-safflower in the treatment of diabetic nephropathy, thus providing a basis for its clinical application and follow-up pharmacologic research. However, this study was based on dating mining and lacks the reliability of in vivo and in vitro experimental validation.

## Figures and Tables

**Table 1:** Potential effective compounds of peach kernel-safflower.

Molecule name	Oral bioavailability (%)	Drug-like	Degree	Herb	Structure
Quercetin	46.43	0.28	146	Safflower	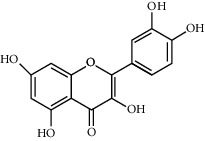
Luteolin	36.16	0.25	96	Peach kernel, safflower	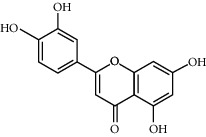
Kaempferol	41.88	0.24	92	Safflower	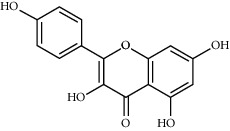
Campesterol	37.58	0.71	61	Peach kernel	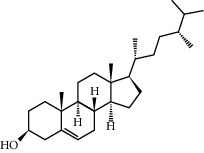
Baicalein	33.52	0.21	53	Safflower	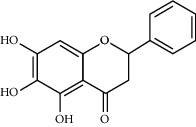
Beta-sitosterol	36.91	0.75	43	Peach kernel, safflower	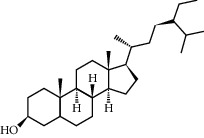
6-Hydroxykaempferol	62.13	0.27	37	Safflower	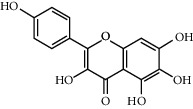
CLR	37.87	0.68	32	Safflower	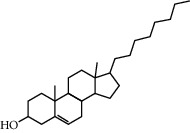
Beta-carotene	37.18	0.58	22	Safflower	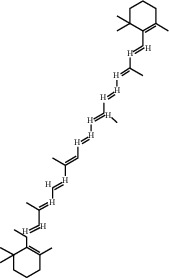
Baicalin	40.12	0.75	21	Safflower	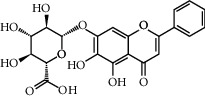
Stigmasterol	43.83	0.76	18	Safflower	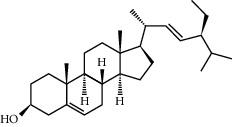
Hederagenin	36.91	0.75	13	Peach kernel	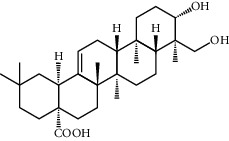
Carthamidin	33.23	0.24	12	Safflower	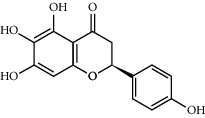
Populoside_qt	108.89	0.2	11	Peach kernel	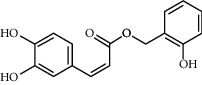
Sitosterol alpha1	43.28	0.78	8	Peach kernel	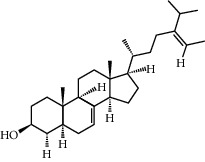
3-O-P-Coumaroylquinic acid	37.63	0.29	7	Peach kernel	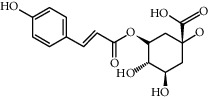
Quercetagetin	45	0.31	5	Safflower	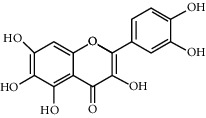
Phytoene	39.56	0.5	3	Safflower
Gibberellin a19	88.6	0.46	2	Peach kernel	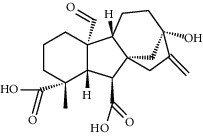
Yuanhuacin	31.83	0.31	1	Peach kernel	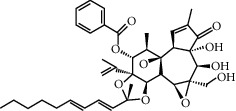
Gibberellin A44	101.61	0.54	1	Peach kernel	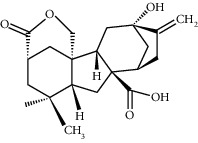
Clionasterol	36.91	0.75	1	Safflower	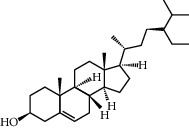
Pyrethrin II	48.35	0.35	1	Safflower	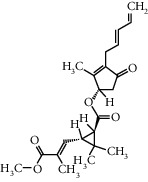

**Table 2:** Protein classes of key targets.

Gene name	Target	Uniprot ID	Protein class	Degree
*VEGFA*	Vascular endothelial growth factor A	P15692	Signaling molecule	54
*IL6*	Interleukin-6	P05231	None	54
*TNF*	Tumor necrosis factor	P01375	Signaling molecule	52
*AKT1*	AKT serine/threonine kinase 1	P31749	Calcium-binding protein; kinase; transfer/carrier protein; transferase	51
*TP53*	Tumor protein p53	P04637	Transcription factor	50

## Data Availability

The data used to support the findings of the study are included within the article.
